# Trends in the use of hematopoietic stem cell transplantation for adults with acute lymphoblastic leukemia in Europe: a report from the Acute Leukemia Working Party of the European Society for Blood and Marrow Transplantation (EBMT)

**DOI:** 10.1007/s00277-019-03771-2

**Published:** 2019-08-07

**Authors:** Sebastian Giebel, Ariane Boumendil, Myriam Labopin, Anouchka Seesaghur, Frederic Baron, Fabio Ciceri, Jordi Esteve, Norbert-Claude Gorin, Bipin Savani, Christoph Schmid, Sally Wetten, Mohamad Mohty, Arnon Nagler

**Affiliations:** 1 Department of Bone Marrow Transplantation and Onco-Hematology, Maria Sklodowska-Curie Institute – Oncology Center, Gliwice Branch, Str. Wybrzeze Armii Krajowej 15, 44-101 Gliwice, Poland; 2 Clinical Hematology and Cellular Therapy Department, Hospital Saint-Antoine, 84 Rue du Faubourg Saint-Antoine, 75012 Paris, France; 3 EBMT Acute Leukemia Working Party Office, Hospital Saint-Antoine, 84 Rue du Faubourg Saint-Antoine, 75012 Paris, France; 4 Amgen Limited, 1 Uxbridge Business Park, Sanderson Road, Uxbridge, London, UB8 1DH UK; 5 Department of Hematology, CHU Sart-Tilman, University of Liège, Avenue de L’Hòpital 1, 4000 Liège, Belgium; 6 Hematology and BMT Unit, IRCCS San Raffaele Scientific Institute, Via Olgettina Milano, 60, Segrate, 20132 Milan, Italy; 7 Hematology Department, IDIBAPS, Hospital Clinic, Carrer del Rosselló, 149, 08036 Barcelona, Spain; 8 Department of Hematology & Transplantation, Vanderbilt University, 2201 West End Ave, Nashville, TN 37235 USA; 9 Department of Hematology and Oncology, Klinikum Augsburg, Ludwig-Maximilians-Universitaet Munich, Stenglinstraße 2, 86156 Augsburg, Germany; 10 Division of Hematology and Bone Marrow Transplantation, Chaim Sheba Medical Center, Tel-HaShomer, Derech Sheba 2, Ramat Gan, Israel

**Keywords:** Acute lymphoblastic leukemia, Allogeneic hematopoietic stem cell transplantation, Autologous hematopoietic stem cell transplantation, Incidence

## Abstract

Hematopoietic stem cell transplantation (HSCT) is considered an effective way to prevent relapse in adults with acute lymphoblastic leukemia (ALL). This study aimed to assess general trends in the use of various types of HSCTs performed between 2001 and 2015 in Europe, based on data reported to the European Society for Blood and Marrow Transplantation registry. We also evaluated HSCT rates with respect to ALL incidence in selected countries. Altogether, 15,346 first allogeneic (*n* = 13,460) or autologous (*n* = 1886) HSCTs were performed in the study period. Comparing 2013–2015 and 2001–2003, the number of allogeneic HSCTs performed in first complete remission increased by 136%, most prominently for transplantations from unrelated (272%) and mismatched related donors (339%). The number of HSCTs from matched sibling donors increased by 42%, while the total number of autologous HSCTs decreased by 70%. Increased use of allogeneic HSCT was stronger for Philadelphia chromosome (Ph)-positive (166%) than for Ph-negative ALL (38%) and for patients aged > 55 years (599%) than for younger adults (59%). The proportion of allogeneic HSCT with reduced-intensity conditioning (RIC) increased from 6 to 27%. The age-standardized rates of allogeneic HSCT per ALL incidence varied strongly among countries. Our analysis showed a continued trend toward increased allogeneic HSCT use for adults with ALL, which may be attributed to increasing availability of unrelated donors, wider use of RIC regimens, and improving efficacy of pretransplant therapy, including tyrosine kinase inhibitors for Ph-positive ALL. Allogeneic HSCT remains a major tool in the fight against ALL in adults.

## Introduction

Acute lymphoblastic leukemia (ALL) is one of the most aggressive malignancies. It is usually sensitive to multi-agent chemotherapy, which allows the initial achievement of complete remission (CR) in the majority of cases [1]. Nonetheless, approximately half of the patients achieving initial CR will relapse, which is associated with a very poor prognosis [2]. Allogeneic hematopoietic stem cell transplantation (HSCT) is considered an effective way to prevent relapse. It offers a chance to use myeloablative doses of chemotherapy and/or radiotherapy and may be associated with the beneficial graft-versus-leukemia reaction mediated by T cells of donor origin.

The efficacy of allogeneic HSCT was confirmed in prospective trials conducted in the 1990s, comparing long-term outcome in subgroups defined based on the availability of human leukocyte antigen (HLA)-matched sibling donor (MSD) [3, 4]. In the twenty-first century, with a growing number of registered volunteers, a chance of finding an HLA-compatible unrelated donor (URD) increased markedly [5]. Finally, novel immunosuppressive protocols have been elaborated, allowing for transplantations from partially mismatched related donors (MMRDs), namely haploidentical donors [6].

Unfortunately, HSCT is associated with a significant risk of life-threatening complications, among which infections and graft-versus-host disease are the most frequent causes of death. Despite improvement observed over time, the estimated risk of nonrelapse mortality remains 15% for HSCTs from MSDs and 22% for HSCTs from URDs [7]. Therefore, many national study groups attempt to reduce the indications for HSCT in favor of more intensive conventional dose chemotherapy. A strict evaluation of response based on minimal residual disease (MRD) assessment allows for a more precise identification of patients at lower risk of relapse. Furthermore, new and more active therapeutic options have been developed and introduced into clinical practice in advanced disease, including immunotoxins, bispecific T cell enhancers, and chimeric antigen receptor T cells [8–11]. For ALL with the presence of *t*(9;22), called Philadelphia chromosome (Ph), new generations of tyrosine kinase inhibitors (TKIs) with strong anti-*ABL* activity became available, offering highly effective ways of targeted therapy [12].

Taken together, the role of HSCT in the treatment of adults with ALL is a subject of debate. The goal of the current study was to analyze trends in the use of HSCT for adults with ALL in Europe. Data were analyzed as absolute number of procedures performed in subsequent time periods and relative to ALL incidence in selected countries. Finally, we looked at associations between the use of HSCT and socioeconomic factors.

## Methods

This is a retrospective registry-based analysis on behalf of the Acute Leukemia Working Party (ALWP) of the European Society for Blood and Marrow Transplantation (EBMT).

The EBMT is a voluntary working group of more than 500 transplant centers that are required to report all consecutive HSCTs and follow-ups once a year. The validation and quality control program includes the verification of computer printouts of the entered data, cross-checking with the national registries, and on-site visits of selected teams.

All allogeneic or autologous HSCTs performed in adult patients (≥ 18 years old) with ALL, between 2001 and 2015, were included in the analysis. The number of procedures was reported in subsequent 3-year intervals (2001–2003, 2004–2006, 2007–2009, 2010–2012, and 2013–2015). The analysis included donor type, disease stage at transplantation, ALL subtypes, recipient age, type and intensity of conditioning regimen, and the source of hematopoietic stem cells.

For the selected 11 countries (Denmark, Finland, France, Germany, Italy, Netherlands, Norway, Poland, Russia, Spain, and Sweden), age-standardized rates (per 100 diagnosed ALL cases) of allogeneic HSCT performed in first CR were calculated from 2001 to 2015 for each country in which ALL incidence data were available. ALL diagnosis incidence data were based on publicly available data reported by country-specific population-based cancer registries. This part of the analysis was restricted to patients aged 20 years or more. Allogeneic HSCT rates for the period 2013–2015 were further correlated with the Human Development Index (HDI) and gross domestic product (GDP) per capita. The values of HDI and GDP were obtained from the 2015 Human Development Report, published by the United Nations [13], referring to data from 2014 and 2013. Spearman test was used to evaluate the associations.

## Results

### HSCT numbers according to donor type and disease stage

**Fig. 1:**
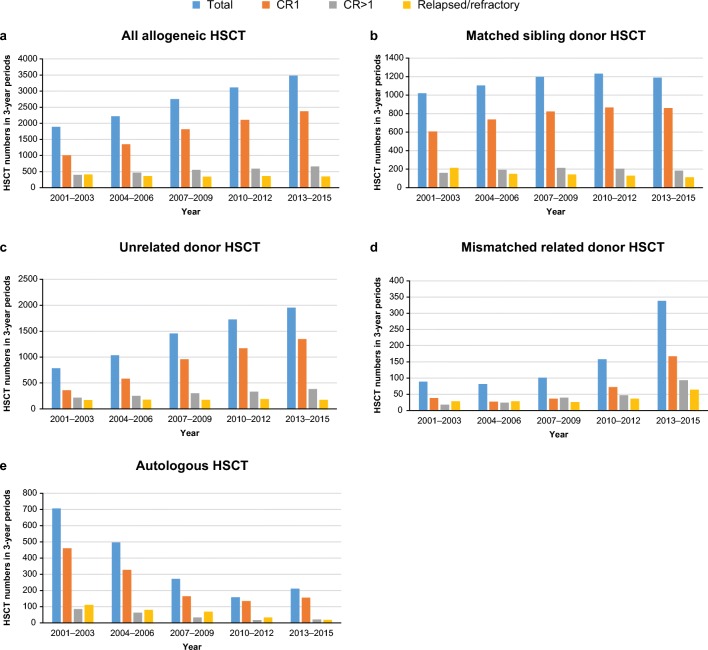
Trends in the use of HSCT according to donor type and disease stage at transplantation. **a** All allogeneic HSCT. **b** Matched sibling donor HSCT. **c** Unrelated donor HSCT. **d** Mismatched related donor HSCT. **e** Autologous HSCT. *CR* complete remission; *CR1* first CR, *CR* > *1* s or subsequent CR; *HSCT* hematopoietic stem cell transplantation

Between 2001 and 2015, 15,346 first HSCT procedures were performed in 32 European countries, including 13,460 allogeneic and 1886 first autologous transplantations. Among allogeneic HSCTs, URDs were used in 6953 cases (52%), followed by MSDs (*n* = 5740; 43%) and MMRDs (*n* = 767; 6%). A continued increase in the total number of allogeneic HSCTs was observed over time, mostly in patients in first CR (Table 1; Fig. 1). Comparing 2013–2015 and 2001–2003, the number of allogeneic HSCTs performed in first CR increased by 136%, most prominently for transplantations from URDs (272%) and MMRDs (339%). In respective periods, the number of HSCTs from MSDs increased by 42%. The number of allogeneic HSCTs for patients treated in second or subsequent CR was stable over time except for MMRD-HSCT, where an increase of 416% was observed. In contrast, the use of autologous HSCTs decreased over time, starting from 707 procedures performed in 2001–2003 to 211 procedures registered in 2013–2015.

**Table 1:** Trends in the use of hematopoietic stem cell transplantation according to donor type and disease status

Donor type	Disease stage at transplantation	Years				
		2001–2003	2004–2006	2007–2009	2010–2012	2013–2015
Allogeneic	Total, *n*	1890	2221	2754	3115	3480
	CR1	1006 (55)	1347 (62)	1816 (67)	2108 (69)	2374 (70)
	CR > 1	398 (22)	468 (22)	556 (20)	588 (19)	660 (19)
	Relapsed/refractory	413 (23)	357 (16)	343 (13)	357 (12)	353 (10)
	Unknown, *n*	73	49	39	62	93
Matched sibling	Total, *n*	1019	1104	1197	1231	1189
	CR1	606 (62)	737 (68)	823 (70)	867 (72)	860 (74)
	CR > 1	161 (16)	192 (18)	215 (18)	206 (17)	183 (16)
	Relapsed/refractory	214 (22)	150 (14)	141 (12)	130 (11)	113 (10)
	Unknown, *n*	38	25	18	28	33
Unrelated	Total, *n*	782	1036	1456	1726	1953
	CR1	362 (48)	583 (57)	957 (67)	1169 (69)	1347 (71)
	CR > 1	219 (29)	252 (25)	302 (21)	335 (20)	384 (24)
	Relapsed/refractory	171 (23)	179 (18)	176 (12)	191 (11)	176 (10)
	Unknown, *n*	30	22	21	31	46
Mismatched related	Total, *n*	89	81	101	158	338
	CR1	38 (45)	27 (34)	36 (36)	72 (46)	167 (52)
	CR > 1	18 (21)	24 (30)	39 (39)	47 (30)	93 (29)
	Relapsed/refractory	28 (33)	28 (35)	26 (26)	36 (23)	64 (20)
	Unknown, *n*	5	2	0	3	14
Autologous	Total, *n*	707	496	272	158	211
	CR1	461 (70)	327 (69)	164 (61)	135 (72)	156 (80)
	CR > 1	85 (13)	64 (14)	34 (13)	18 (10)	21 (11)
	Relapsed/refractory	111 (17)	80 (17)	69 (26)	34 (18)	19 (10)
	Unknown, *n*	50	25	5	13	15

Data are *n* (%) using non-missing data unless specified otherwise. Only first allogeneic or autologous transplantations were included in the analysis

*CR* complete remission; *CR1* first CR, *CR* > *1* s or subsequent CR

Altogether, the proportion of allogeneic HSCTs analyzed by donor type changed in favor of URD (41% versus 56% URD-HSCTs among all transplantations between 2001 and 2003 versus 2013 and 2015) and MMRD (5% versus 10%).

### HSCT numbers by disease subtype

Data regarding the presence of Ph status were available for approximately 55% of the recipients of allogeneic HSCT with B cell precursor ALL. In these patients, the absolute number of allogeneic transplantations increased over time for both Ph-negative and Ph-positive ALL; however, the increase was more pronounced for Ph-positive versus Ph-negative disease (166% versus 38% from 2001–2003 to 2013–2015; Table 2). The proportion of transplants for Ph-positive ALL among allogeneic HSCT recipients with known karyotype increased from 31 to 40%. In parallel, the number of autologous HSCTs decreased by 78% and 5% for Ph-negative and Ph-positive ALL, respectively. Notably, between 2013 and 2015, patients with Ph-positive ALL constituted 47% of autologous HSCT recipients compared with 17% between 2001 and 2003.

**Table 2:** Trends in the use of hematopoietic stem cell transplantation according to disease subtype

Donor type	Disease subtype	Ph status	Years				
			2001–2003	2004–2006	2007–2009	2010–2012	2013–2015
Allogeneic	Total, *n*		1890	2221	2754	3115	3480
Allogeneic	B cell precursor	Total	1211 (73)	1535 (73)	1959 (77)	2166 (75)	2502 (78)
		Ph-negative	327 (27)	343 (22)	342 (17)	380 (17)	452 (18)
		Ph-positive	377 (31)	417 (27)	729 (37)	812 (37)	1003 (40)
		Unknown, *n*	507	775	888	974	1047
	T cell precursor		403 (24)	539 (26)	591 (23)	704 (24)	720 (22)
	Other		42 (3)	35 (2)	3 (0)	6 (0)	4 (0)
	Unknown, *n*		234	112	201	239	254
Autologous	Total, *n*		707	496	272	200	211
	B cell precursor	Total	381 (59)	259 (55)	165 (64)	110 (60)	127 (65)
		Ph-negative	79 (21)	45 (17)	30 (18)	12 (11)	17 (13)
		Ph-positive	63 (17)	37 (14)	50 (30)	44 (40)	60 (47)
		Unknown, *n*	239	177	85	54	50
	T cell precursor		247 (38)	206 (44)	94 (36)	74 (40)	66 (34)
	Other		20 (3)	6 (1)	0 (0)	0 (0)	1 (1)
	Unknown, *n*		59	25	13	16	17

Data are *n* (%) using non-missing data unless specified otherwise. Only first allogeneic or autologous transplantations were included in the analysis

*Ph* Philadelphia chromosome

Data on immune subtype were available for 92% of the patients treated with allogeneic HSCT and 93% of those treated with autologous HSCT. Similar trends were observed for both B cell precursor and T cell precursor ALL (Table 2). The number of allogeneic HSCTs was continuously increasing, while the number of autologous HSCTs was decreasing over time regardless of the immune subtype.

### HSCT and age

Between 2001 and 2003, patients older than 55 years constituted 5% of all adults treated with allogeneic HSCT and 13% of those treated with autologous HSCT, while between 2013 and 2015, the proportions were 18% and 27%, respectively (Table 3). The most prominent increase was observed for URD-HSCT (by 1415%) and MMRD-HSCT (by 2300%). The 55 years age limit is most frequently used by the European ALL study groups to define patients’ eligibility for intensive chemotherapy and myeloablative transplant procedures [14].

**Table 3:** Trends in the use of hematopoietic stem cell transplantation according to donor type and recipient age category

Donor type	Age (years)	Years				
		2001–2003	2004–2006	2007–2009	2010–2012	2013–2015
Total allogeneic	18–55	1801 (95)	2069 (93)	2428 (88)	2679 (86)	2858 (82)
	> 55	89 (5)	152 (7)	326 (12)	436 (14)	622 (18)
Matched sibling	18–55	958 (94)	1027 (93)	1054 (88)	1085 (88)	1009 (85)
	> 55	61 (6)	77 (7)	143 (12)	146 (12)	180 (15)
Unrelated	18–55	756 (97)	961 (93)	1280 (88)	1452 (84)	1559 (80)
	> 55	26 (3)	75 (7)	176 (12)	274 (16)	394 (20)
Mismatched related	18–55	87 (98)	81 (100)	94 (93)	142 (90)	290 (86)
	> 55	2 (2)	0 (0)	7 (7)	16 (10)	48 (14)
Autologous	18–55	614 (87)	420 (85)	211 (78)	146 (73)	153 (73)
	> 55	93 (13)	76 (15)	61 (22)	54 (27)	58 (27)

Data are *n* (%). Only first allogeneic or autologous transplantations were included in the analysis

### Conditioning regimens and stem cell source

Several aspects of the transplantation procedure, including the type of conditioning and source of hematopoietic stem cells, were analyzed for patients treated with allogeneic HSCT in first CR (Table 4). The proportion of transplantations with reduced-intensity conditioning (RIC) increased from 6% in 2001–2003 to 26% in 2010–2012 [15]. Among myeloablative regimens, the use of total body irradiation (TBI) was predominant and stable over time, accounting for approximately 80% of all procedures. In contrast, chemotherapy-based regimens were more frequently used in the reduced-intensity setting, and their proportion increased from 53% in 2001–2003 to 84% in 2013–2015.

**Table 4:** Type of conditioning and stem cell sources for allogeneic hematopoietic stem cell transplantation in first complete remission

		Years				
		2001–2003	2004–2006	2007–2009	2010–2012	2013–2015
Intensity of conditioning	Total, *n*	1006	1347	1816	2108	2374
	Myeloablative	901 (94)	1184 (89)	1537 (86)	1678 (81)	1689 (73)
	Reduced	60 (6)	146 (11)	256 (14)	394 (19)	625 (27)
	Unknown, *n*	45	17	23	36	60
Myeloablative conditioning	Total, *n*	901	1184	1537	1678	1689
	Chemotherapy based	118 (13)	166 (14)	272 (18)	313 (19)	393 (23)
	TBI based	783 (87)	1015 (86)	1263 (82)	1365 (81)	1294 (77)
	Unknown, *n*	0	3	2	0	2
Reduced-intensity conditioning	Total, *n*	60	146	256	394	625
	Chemotherapy based	32 (53)	89 (63)	182 (71)	311 (79)	525 (84)
	TBI based	28 (47)	53 (37)	74 (29)	83 (21)	99 (16)
	Unknown, *n*	0	4	0	0	1
Stem cell source	Total, *n*	1006	1347	1816	2108	2374
	Bone marrow	385 (38)	415 (31)	410 (23)	448 (21)	372 (16)
	Peripheral blood	616 (61)	926 (69)	1401 (77)	1652 (78)	1994 (84)
	Cord blood	3 (0)	6 (0)	4 (0)	4 (0)	4 (0)
	Unknown, *n*	2	0	1	4	5

Data are *n* (%) using non-missing data unless specified otherwise

*TBI* total body irradiation

Peripheral blood was the predominant source of stem cells, accounting for 61% of the allogeneic HSCT procedures in 2001–2003, with an increase up to 84% in 2013–2015. In the most recent period, 16% of transplants were performed using bone marrow as a source of stem cells when compared to 38% in the period 2001–2003. Cord blood use was limited to less than 1% throughout the 15-year study period.

### HSCT rates per ALL incidence

**Fig. 2:**
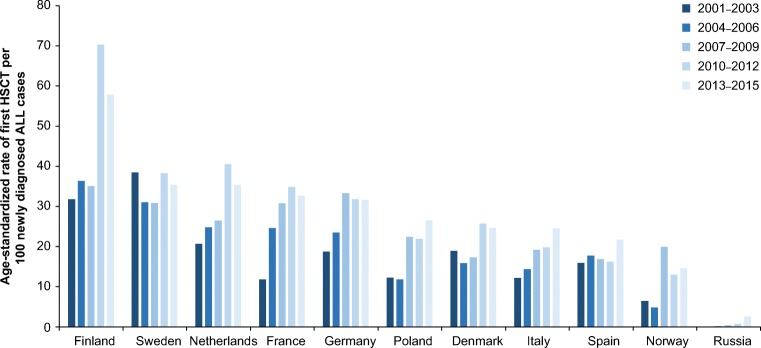
Age-standardized rates of first allogeneic HSCT performed in first complete remission for ALL in selected European countries. *ALL* acute lymphoblastic leukemia, *HSCT* hematopoietic stem cell transplantation

Age-standardized rates of first allogeneic HSCT performed in first CR, calculated per 100 diagnosed ALL incidence cases in patients aged 20 years and above, varied substantially among the analyzed countries (Fig. 2). The highest rates and most prominent increase in recent period were observed in Finland, followed by the Netherlands and Sweden; the lowest rates were noted in Russia. In several countries, including Germany and Poland, a marked increase was observed between 2007 and 2009 compared with the preceding periods, followed by a plateau in 2010–2015.

No statistically significant associations could be demonstrated for HSCT rates with either HDI (Spearman correlation = 0.21; *p* = 0.54) or GDP per capita (Spearman correlation = − 0.08; *p* = 0.81).

## Discussion

This comprehensive analysis on the activity of transplantation for ALL showed a marked increase in the number of allogeneic HSCTs performed during a 15-year period (2001–2015), especially in first CR, as a consolidation strategy aimed to decrease relapse risk. This increased transplant activity was based on diverse reasons, such as (a) the use of highly effective TKIs in Ph-positive ALL, resulting in a significantly high proportion of patients achieving a major response that allowed bridging to allogeneic HSCT; (b) the wider availability of unrelated donors that currently constitute the main donor source; and (c) the increased utility of RIC allogeneic HSCT, which expands the potential benefit of transplantation to the older ALL population.

Modern therapeutic protocols differ substantially for patients with Ph-positive and Ph-negative ALL. The presence of *t*(9;22) was traditionally considered a very-high-risk feature—associated with a lower chance of achieving CR and a very high probability of relapse [16]. Allogeneic HSCT provided the only reasonable chance of cure. The situation changed with the introduction of TKIs, which became widely available in the middle of the last decade. As demonstrated by studies comparing combined imatinib + chemotherapy with historical controls using chemotherapy alone, CR rates increased, followed by prolonged relapse-free survival [17]. However, without allogeneic HSCT, a majority of the patients ultimately relapse [18]. According to a study by Chalandon et al., allogeneic HSCT is still associated with significant benefit in terms of the overall survival in adults with Ph-positive ALL, treated initially with the combination of imatinib and reduced-dose chemotherapy [19]. The outcome may further be improved when TKIs are used as posttransplant maintenance [20]. Results of our retrospective database analysis indicate that the number of allogeneic HSCTs in first CR almost tripled for Ph-positive ALL within the 15-year period. Clearly, the addition of imatinib to standard clinical practice improved the quality of response and reduced the risk of early relapse, therefore increasing the chance for patients to be treated with transplantation. In parallel, increasing availability of unrelated donors allowed for identification of HLA-compatible donors for the majority of patients in most European countries. More recently, the implementation of highly efficacious protocols of immunosuppression, such as the use of cyclophosphamide early after transplantation from haploidentical donors, offers the availability of a potential adequate donor to almost all patients in need of allogeneic HSCT. Hence, it may be anticipated that the number of transplants for adults with Ph-positive ALL will further increase. On the other hand, there are attempts to improve results of conventional-dose treatment regimens by adding second-generation (dasatinib, nilotinib) or third-generation (ponatinib) TKIs to first-line treatment. In a study by the European Working Group for Adult ALL, dasatinib in combination with chemotherapy was offered to patients older than 55 years, who are usually not candidates for myeloablative HSCT [21]. Despite a 65% rate of major molecular responses, most patients relapsed within 5 years, leading to an overall survival probability of 35%. Jabbour et al. reported the use of ponatinib in combination with intensive chemotherapy for younger adults with Ph-positive ALL [22]. The 2-year probability of event-free survival was 81%, although allogeneic HSCT was offered to only nine out of 37 patients. It must be stressed, however, that the follow-up of the study was relatively short, while long-term safety of ponatinib remains a matter of concern. Finally, with increasing efficacy of first-line treatment, it appeared that autologous HSCT followed by TKI maintenance may be a valuable alternative to allogeneic HSCT, especially for patients achieving molecular remission [23, 24]. According to our current study, the use of autologous HSCT for patients with Ph-positive ALL was low and rather stable over time; however, we speculate that some increase may be anticipated in the future.

In contrast to Ph-positive ALL, treatment algorithms for adults with Ph-negative disease are still based mainly on chemotherapy. In recent years, the chemotherapy protocols have been intensified following the pediatric experience in younger adults. The indications for allogeneic HSCT are usually based on risk stratification, with special attention put on the evaluation of response at the level of MRD. According to results of a study conducted by a French group using a pediatric-inspired protocol, patients who achieve an MRD level below 10^−3^ after induction chemotherapy do not necessarily benefit from allogeneic HSCT, regardless of the presence of conventional high-risk features [25]. Results of our study demonstrated a trend toward an increasing number of allogeneic HSCTs, although much less pronounced compared with Ph-positive ALL. Therefore, it may be speculated that the increasing availability of donors may be partially counterbalanced by restricted indications for HSCT in patients achieving MRD negativity. In the future, wide introduction of novel humoral and cellular immunotherapeutic approaches may change the landscape. The use of rituximab in first-line treatment has already been demonstrated to increase the probability of leukemia-free survival of patients with CD20-positive ALL [26]. Drugs like blinatumomab or inotuzumab ozogamicin are highly effective in the relapsed/refractory B cell precursor ALL setting and may serve as a bridge, enabling successful allogeneic HSCT [8, 9]. On the other hand, when used frontline, they may increase the chance of MRD eradication, further limiting the role of transplant procedures.

ALL in elderly patients is associated with very poor prognosis due to the high risk of chemotherapy-related complications, hence the need for dose intensity reduction [27]. These patients are usually not candidates for transplantations with myeloablative preparative regimens. In late 1990s, RIC regimens were developed and introduced, allowing for wider application of allogeneic HSCT to older patients, including those with ALL [28]. This is reflected in the results of our study showing a seven-fold increase in the total number of allogeneic HSCT and 15-fold increase in the number of URD-HSCT for patients aged above 55 years. In similar proportions, the number of reported RIC procedures increased over time. Following general demographical trends toward increasing life expectancy, it may be anticipated that the number of older patients in need for allogeneic HSCT, and consequently, the number of procedures, will further grow in the future.

Numerous retrospective studies, including the recent analysis by our group [7], indicate that myeloablative TBI-based preparative regimens are more effective than chemotherapy-based conditioning, resulting in decreased risk of relapse and improved leukemia-free survival. Results of the current study confirm the predominant role of TBI in myeloablative setting, which was stable over time. It may be speculated, however, that with the development of new reduced-toxicity chemotherapy-based protocols, the landscape may change in the future. Encouraging results have already been reported for preparative regimens using thiotepa as a backbone [29]. Peripheral blood was the predominant source of stem cells over the entire study period showing a continuous increase over time. This phenomenon has been already reported for allogeneic HSCT in other indications [30].

Allogeneic HSCT is considered one of the most expensive medical procedures. The costs of URD-HSCT, most frequently used for patients with ALL, are particularly high and were calculated as US dollars (USD) 161,000 in Sweden and USD 152,000 in the Netherlands [31, 32]. According to the analysis by Stranges et al., HSCTs were procedures with the most rapidly increasing costs of hospital stay in the USA between 2004 and 2007 [33]. The use of HSCT for adults with ALL varies greatly among countries, which may reflect that the availability of these procedures may be related to economic issues. However, results of our analysis do not show a clear correlation between HSCT rates and socioeconomic indices. Our findings suggest that the use of HSCT may be dependent on national guidelines, and the observed variation reflects heterogeneity of treatment approaches among countries. It should also be mentioned that the costs of novel drugs and cellular therapies may exceed the costs of HSCT.

Our study had some limitations, including any assumptions made regarding ALL incidence for the specified time period and possible variation in reporting to the EBMT registry from different countries over time. Despite that, we conclude that allogeneic HSCT remains a major tool in the fight against ALL in adults.

## Data Availability

The datasets generated during and/or analyzed during the current study are available from the corresponding author on reasonable request.
